# Kir6.2-containing ATP-sensitive K^+^ channel is required for cardioprotection of resveratrol in mice

**DOI:** 10.1186/1475-2840-13-35

**Published:** 2014-02-05

**Authors:** Ren-Hong Du, Ting Dai, Wen-Jing Cao, Ming Lu, Jian-hua Ding, Gang Hu

**Affiliations:** 1 Department of Pharmacology, Nanjing Medical University, 140 Hanzhong Road, Nanjing, Jiangsu 210029, P.R. China

**Keywords:** Resveratrol, ATP-sensitive potassium channel, Kir6.2, Myocardial Ischemia/reperfusion, 5′-AMP-activated protein kinase

## Abstract

**Background:**

Resveratrol is a natural compound that affects energy metabolism and is also known to possess an array of cardioprotective effects. However, its overall effects on energy metabolism and the underlying mechanism involved in cardioprotection require further investigation. Herein we hypothesize that ATP-sensitive potassium (K-ATP) channels as molecular sensors of cellular metabolism may mediate the cardioprotective effects of resveratrol.

**Methods:**

Kir6.2 knockout, Kir6.1 heterozygous and wild-type (WT) mice were subjected to ischemia/reperfusion injury and were injected with resveratrol (10 mg/kg, i.p). Myocardial infarct size, serum lactate dehydrogenase (LDH) and creatine kinase (CK) activities were determined. Neonatal cardiomyocytes were used in *in vitro* assays to investigate the underlying mechanism of resveratrol in cardioprotection.

**Results:**

Resveratrol treatment significantly reduced myocardial infarct size and serum LDH and CK activity and inhibited oxygen-glucose deprivation/reoxygenation – induced cardiomyocyte apoptosis in WT and Kir6.1 heterozygous mice, but Kir6.2 deficiency can abolish the cardioprotective effects of resveratrol *in vivo* and *in vitro*. We further found that resveratrol enhanced 5′-AMP-activated protein kinase (AMPK) phosphorylation and promoted the association of AMPK with Kir6.2. Suppression of AMPK attenuated and activation of AMPK mimicked the cardioprotective effects of resveratrol in cardiomyocytes. Notably, Kir6.2 knockout also reversed the cardioprotection of AMPK activator.

**Conclusions:**

Our study demonstrates that resveratrol exerts cardioprotective effects through AMPK -Kir6.2/K-ATP signal pathway and Kir6.2-containing K-ATP channel is required for cardioprotection of resveratrol.

## Background

Resveratrol (3, 4′, 5-trihydroxy-trans-stilbene) is a naturally existing polyphenol phytoalexin originally isolated from the roots of white hellebore and later found in a variety of fruits, vegetables, and grape skins [1]. Resveratrol has extensive biological and pharmacological effects, such as anti-atherosclerosis [2], anti-inflammation [3], anti-oxidation [4], and anti-cancer activities [5]. Recent studies have demonstrated that resveratrol can protect the heart from ischemia/reperfusion (I/R) injury [6]. Suppression of superoxide levels, activation of potassium channels and translocation of GSK-3β have been proposed to mediate the cardioprotective effects of resveratrol [6,7]. However, the underlying mechanisms of resveratrol against myocardial I/R injury have not yet been fully explored.

ATP sensitive potassium (K-ATP) channels are hetero-octamers composed of pore-forming Kir6.x (6.1 or 6.2) subunits and sulfonylurea receptor (SUR1 or SUR2) regulatory subunits, regulated by intracellular ATP and ADP concentrations [8] K-ATP channels are highly expressed in cardiomyocytes and play crucial roles in ischemic preconditioning (IPC) [9,10]. Potassium channel openers mimicked the cardioprotection and the K-ATP channel blocker abolished the effect of IPC [11,12]. In addition, Kir6.2 knockout abolished Ischemia- and diazoxide- induced preconditioning [10,13]. These results indicate that K-ATP channel is a key target for cardioprotection. Importantly, resveratrol exerts multiple cardioprotective effects similar to those associated with energy metabolism, and K-ATP channels couple cell metabolism to cell membrane potential. However, the link between resveratrol and K-ATP channel in myocardial I/R injury remains unclear. Therefore, it is intriguing to determine whether K-ATP channel is involved in the cardioprotection of resveratrol.

In the present study, Kir6.2 knockout mice (Kir6.2^-/-^) and Kir6.1 heterozygote mice (Kir6.1^+/-^) were used to define the roles of Kir6.1- and Kir6.2- containing K-ATP (Kir6.1/K-ATP and Kir6.2/K-ATP) channels in resveratrol-mediated protection against myocardial I/R injury. Our results demonstrate that Kir6.2/K-ATP channels are required for resveratrol-mediated protection against myocardial I/R injury.

## Methods

### Animals and reagents

Kir6.2 knockout mice donated by Professor Miki (Kobe University, Japan), Kir6.1 heterozygous mice (purchased from MMRRC Company) and wild-type (WT) mice were bred and maintained in the Animal Resource Centre of the Faculty of Medicine, Nanjing Medical University. Knockout of the Kir6.2 gene was confirmed by RT-PCR. Littermate mice were used as WT controls, as previously described [14]. Age-matched adult male mice (3 months old) were utilized for the experiments. All experimental procedures were conducted in strict accordance with the National Institutes of Health Guide for the Care and Use of Laboratory Animals and all animals were treated according to protocols approved by IACUC (Institutional Animal Care and Use Committee of Nanjing Medical University).

Resveratrol was purchased from Cayman Chemical Company (Ann Arbor, MI), and dissolved in either ethanol or DMSO. Evans blue, triphenyl tetrazolium chloride (TTC), compound C and 5-aminoimidazole-4-carboxamide- ribonucleoside (AICAR) were purchased from Sigma Chemical Company.

### Myocardial ischemia-reperfusion (I/R) protocol

Mice were anesthetized with 2% isoflurane. The skin on the neck was opened and the trachea was cut down to guide the placement of an end tracheal tube. The animals were mechanically ventilated with room air supplemented with oxygen using an end tracheal tube and a rodent ventilator. During the operation, body temperature was maintained at 37°C using a heat lamp. After a left thoracotomy was performed in the fourth intercostal space, the pericardium was opened to expose the heart. A 6-0 silk suture slipknot was passed around the proximal portion of the left anterior descending artery (LAD) at a point two thirds of the way between its origin near the pulmonary conus and the cardiac apex. Regional ischemia was confirmed by visual inspection of the discoloration of the heart. Mice were subjected to 30 min of ligation, followed by reperfusion by releasing the slipknot. Successful reperfusion was confirmed by visualization of the return of a bright red color in the previously discolored region. Sham-operated mice (Sham) underwent the same surgical procedure but without the ligation of the LAD. It has been demonstrated that the cardioprotective effect of resveratrol was detectable at 5 mg/kg in isolated rat hearts [15,16]. According to the dose of resveratrol used in rats, we performed all experiments with 10 mg/kg resveratrol in our study, because the resveratrol 10 mg/kg is the most effective dose that can protect against myocardial ischemia/reperfusion injury in mice, whereas at a lower dose of 1 mg/kg resveratrol or at higher dose of 100 mg/kg resveratrol was found to be only partially protective. In the experiment, resveratrol (10 mg/kg) or 15% ethanol vehicle (10 ml/kg) was intraperitoneally injected into the mice at min 60 before the ligation.

### Determination of myocardial infarct size

At the end of 24-hour reperfusion, mice were re-anesthetized, and LAD was re-occluded in the same location as before. Evans blue (1 ml of the 2% solution) was injected into the left ventricular cavity to perfuse the non-ischemic area, delineating the myocardium as dark blue. The area at risk (AAR), the portion of the left ventricle supplied by the previously occluded coronary artery, was determined by the absence of blue. The heart was then excised, rinsed of excess blue dye, trimmed of atrial tissue, and sliced into 1-mm-thick cross sections. After being incubated in 1% TTC at 37°C for 25 min to stain the viable myocardium red, the slices were then fixed with 10% formaldehyde. The sizes of the infarct area and risk area were determined by individuals blinded to the experimental groups using computer-assisted planimetry (NIH software Image 1.0). Infarct size was calculated as a percentage of AAR to indirectly assess the degree of myocardial damage.

### Cultures of neonatal cardiomyocytes

Ventricular myocytes were prepared from embryonic mice (E19-20) by trypsin digestion. Briefly, ventricles of mice were aseptically removed immediately after decapitation. Isolated hearts were pooled, minced, and digested at 37°C with trypsin. Dissociated cells were plated for 60 minutes onto 100-mm-diameter dishes to reduce the number of nonmyocardial cells, in the presence of Dulbecco’s modified Eagle’s medium (DMEM) (GIBCO) supplemented with 10% fetal serum (Hyclone Laboratories, Logan, UT). After being centrifuged at a low speed, cells were plated at a low density (4 × 10^5^ cells mL^-1^) in 24-well plates, and incubated for 24 h at 37°C in a humidified atmosphere of 95%O_2_/5%CO_2_. The culture medium was then changed to DMEM containing 10% fetal serum and 0.1 mM 5-BrdU.

### Oxygen-glucose deprivation/reoxygenation (OGD/R) experimental protocol

Cardiomyocytes were prepared as described above, and used on day 4 of culture. Resveratrol (compound C or AICAR) was added to the medium for 30 min before OGD and maintained in the medium during the subsequent twenty-four hours of recovery period. For OGD, the culture medium was replaced by thorough exchange with glucose-free medium containing 25 mM sucrose, and then the cells were incubated in a humidified atmosphere of 5% CO_2_, 94 N_2_% and 1% O_2_. Twenty four hours after reoxygenation, cells were fixed with 4% formaldehyde in PBS for immunocytochemistry.

### Hoechst staining

Twenty four hours after OGD, cardiomyocyte monolayer was fixed and stained with Hoechst 33342 (Sigma) to quantify apoptotic cells. The morphological features of apoptosis (cell shrinkage, chromatin condensation, and fragmentation) were monitored by Nikon Optical TE2000-S inverted fluorescence microscope. At least 400 cells from 12 randomly selected fields per well were evaluated to determine the number and percentage of cells exhibiting apoptosis, and each treatment was performed in duplicate.

### Levels of CK and LDH in serum and culture medium

Creatine kinase (CK) and lactate dehydrogenase (LDH) in serum and culture media were determined using a commercially available kit (Nanjing Jiancheng Bioengineering Institute, Nanjing, China).

### Coimmunoprecipitation

The lysates from cardiomyocytes were immunoprecipitated with anti-AMPK antibody followed by protein A/G plus agarose (Santa Cruz Biotechnology). After washing, bound proteins were eluted from the beads and analyzed by immunoblot for Kir6.2.

### Western blot analysis

The left ventricle was homogenized in lyses buffer (Beyotime, China) and protein concentration was determined by the Bradford assay (Bio-Rad, Hercules, CA, USA). Proteins were separated on 10% Tris-HCl polyacrylamide gels (Bio-Rad) and transferred to a PVDF membrane. After blocking, the blots were incubated with anti-phospho-AMPK (2531, Cell Signaling Technology, Beverly, MA, USA), anti-AMPK (2532, Cell Signaling Technology, Beverly, MA, USA), anti-Kir6.2 (sc-20809, Santa Cruz Biotechnology) and anti-Kir6.1 (sc-11224, Santa Cruz Biotechnology) in TBST overnight at 4°C, and then with horseradish peroxidase (HRP) conjugated secondary antibodies. Immunoreactive bands were detected by enhanced chemiluminescence (ECL) plus detection reagent (Pierce, Rockford, IL, USA), and analyzed using an Omega 16ic Chemiluminescence Imaging System (Ultra-Lum, CA, USA).

### Statistical analysis

Data are presented as mean ± SEM. The significance of the difference with different treatments and genotypes was determined by one-way or two-way ANOVA, followed by the Tukey’s post hoc test. Differences were considered significant at P < 0.05.

## Results

### Resveratrol increases the expression of Kir6.2 subunits in heart following I/R injury

**Figure 1:**
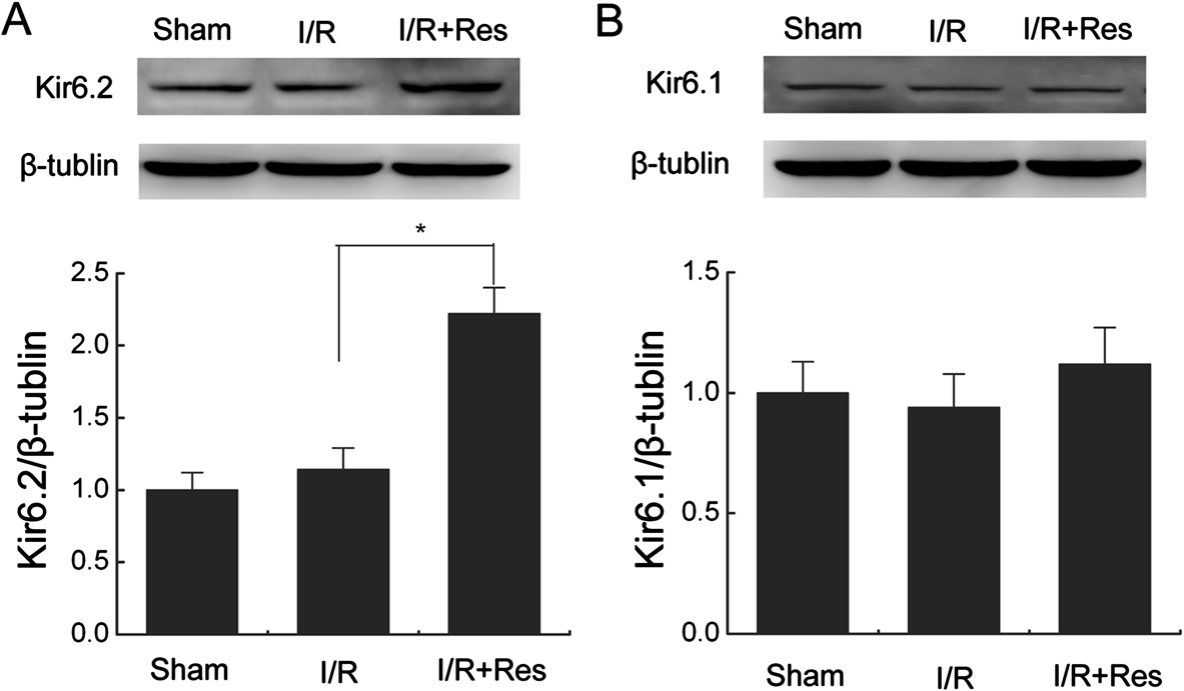
**Resveratrol increased the expression of Kir6.2 subunits in heart following ischemia/reperfusion injury. (A-B)** Representative immunoblots and quantification of myocardial protein extracts for analysis of Kir6.2 **(A)** or Kir6.1 protein **(B)** (n = 5/group). Data are represented as means ± SEM. ^*^P < 0.05. I/R = ischemia/reperfusion; Res = resveratrol.

To investigate the roles of K-ATP channels in the cardioprotective effects of resveratrol, we first examined the expression of Kir6.1 and Kir6.2 subunits in the left ventricle of WT mice after I/R injury with or without resveratrol treatment. Western blotting showed that protein expression of Kir6.1 and Kir6.2 were not changed after I/R injury. Resveratrol (10 mg/kg) induced a significant increase of Kir6.2 subunits expression in the left ventricle of WT mice after I/R injury (2.22 ± 0.18 vs. 1.14 ± 0.15, *P < 0.05,* Figure 1A). However, the expression levels of Kir6.1 subunits were not affected by resveratrol treatment (1.12 ± 0.15 vs. 0.94 ± 0.14, *P > 0.05,* Figure 1B). These results suggest that Kir6.2/K-ATP channels may be involved in the cardioprotection of resveratrol.

### Kir6.2 knockout abolishes the protection of resveratrol against I/R injury in mice

**Figure 2:**
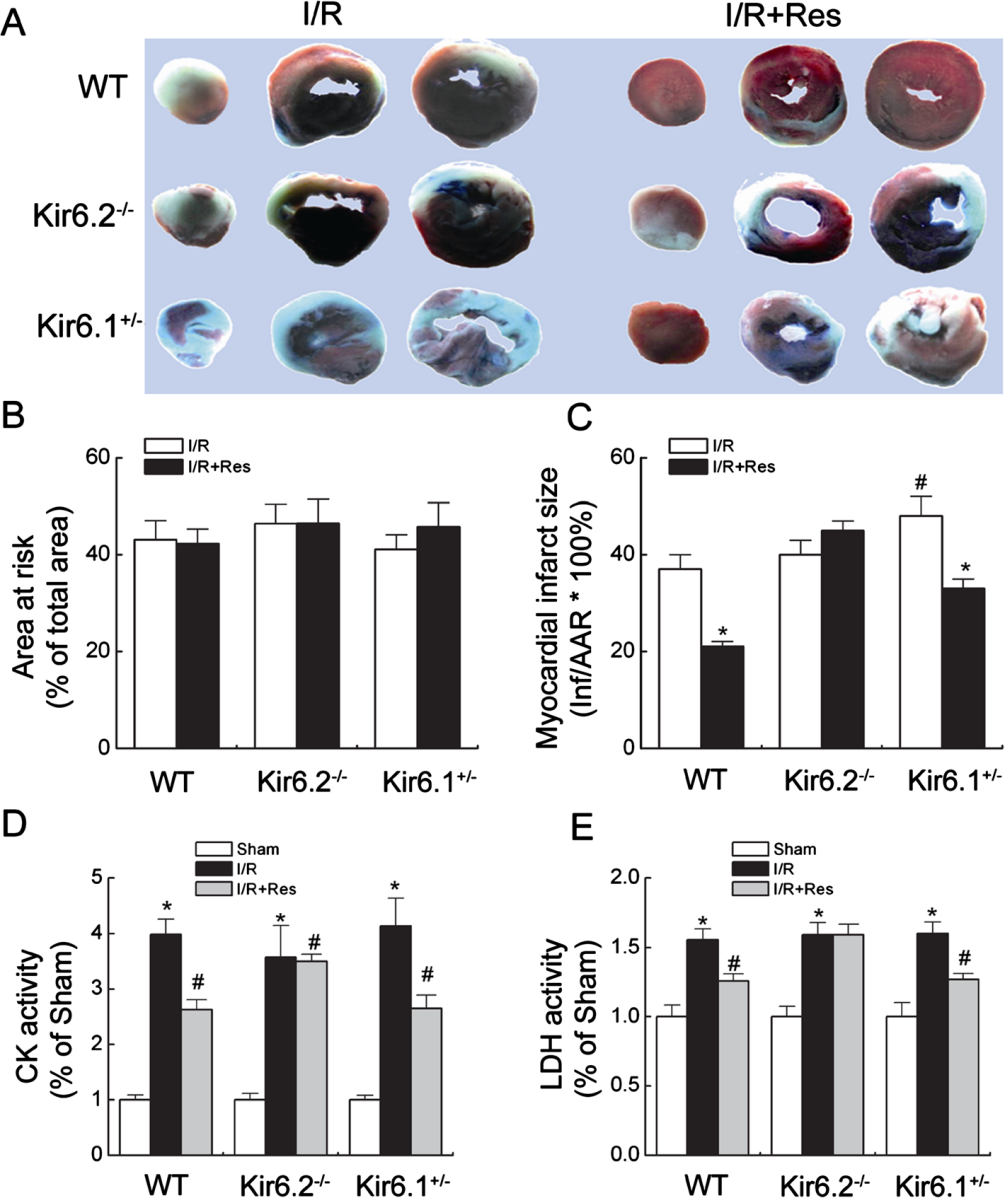
**Kir6.2 knockout abolished the protection of resveratrol against ischemia/reperfusion injury in mice. (A)** Representative photograms of myocardial slices stained by Evans-blue/TTC. **(B)** Myocardial area at risk expressed as percentage of total area. **(C)** Myocardial infarct size expressed as percentage of AAR (n = 8/group). ^*^P < 0.01 *vs*. corresponding I/R group; ^#^*P <* 0.05 *vs*. WT I/R group. **(D-E)** Cardiac injury determined by serum CK **(D)** and LDH activity **(E)** (n = 8/group). Data are represented as means ± SEM. ^*^P < 0.01 *vs*. corresponding sham group; ^#^*P <* 0.01 *vs*. corresponding I/R group.

To further determine the roles of K-ATP channels in resveratrol mediated protection against myocardial I/R injury, we compared myocardial infarct size in WT, Kir6.2 knockout (Kir6.2^-/-^) and Kir6.1 heterozygote mice (Kir6.1^+/-^). Figure 2A showed representative photograms of myocardial slices stained with Evans blue dye to AAR and TTC to delineate infarct areas. There were no significant differences in the size of the AAR among three genotypic mice with or without resveratrol treatment (Figure 2B). The infarct sizes were similar between WT mice and Kir6.2^-/-^ mice subjected to I/R injury (37% ± 3% vs 40% ± 3%). Kir6.1^+/-^ mice exhibited the greater infarct size than WT mice after I/R injury (48% ± 4% vs 37% ± 3%, Figure 2C). Resveratrol significantly decreased the infarct size from 37% ± 3% to 21% ± 1% in WT mice and from 48% ± 4% to 33% ± 2% in Kir6.1^+/-^ mice, respectively. However, resveratrol failed to reduce the infarct size in Kir6.2^-/-^ mice (45% ± 2%, Figure 2C).

In addition, serum LDH and CK activities, markers for the confirmation of myocardial injury, were determined after I/R injury. Serum CK and LDH activities were significantly increased in WT mice, Kir6.2^-/-^ mice and Kir6.1^+/-^ mice subjected to I/R injury. Treatment with resveratrol markedly suppressed I/R-induced serum CK and LDH elevation in WT and Kir6.1^+/-^ mice (Figure 2D-E). Similarly, resveratrol did not substantially reduce serum CK and LDH elevation in Kir6.2^-/-^ mice after I/R injury (Figure 2D-E). These results indicate that protection of resveratrol against I/R injury is dependent on Kir6.2/K-ATP channels, rather than Kir6.1/K-ATP channels.

### Kir6.2 deficiency reverses protection of resveratrol against OGD/R injury in cardiomyocytes

**Figure 3:**
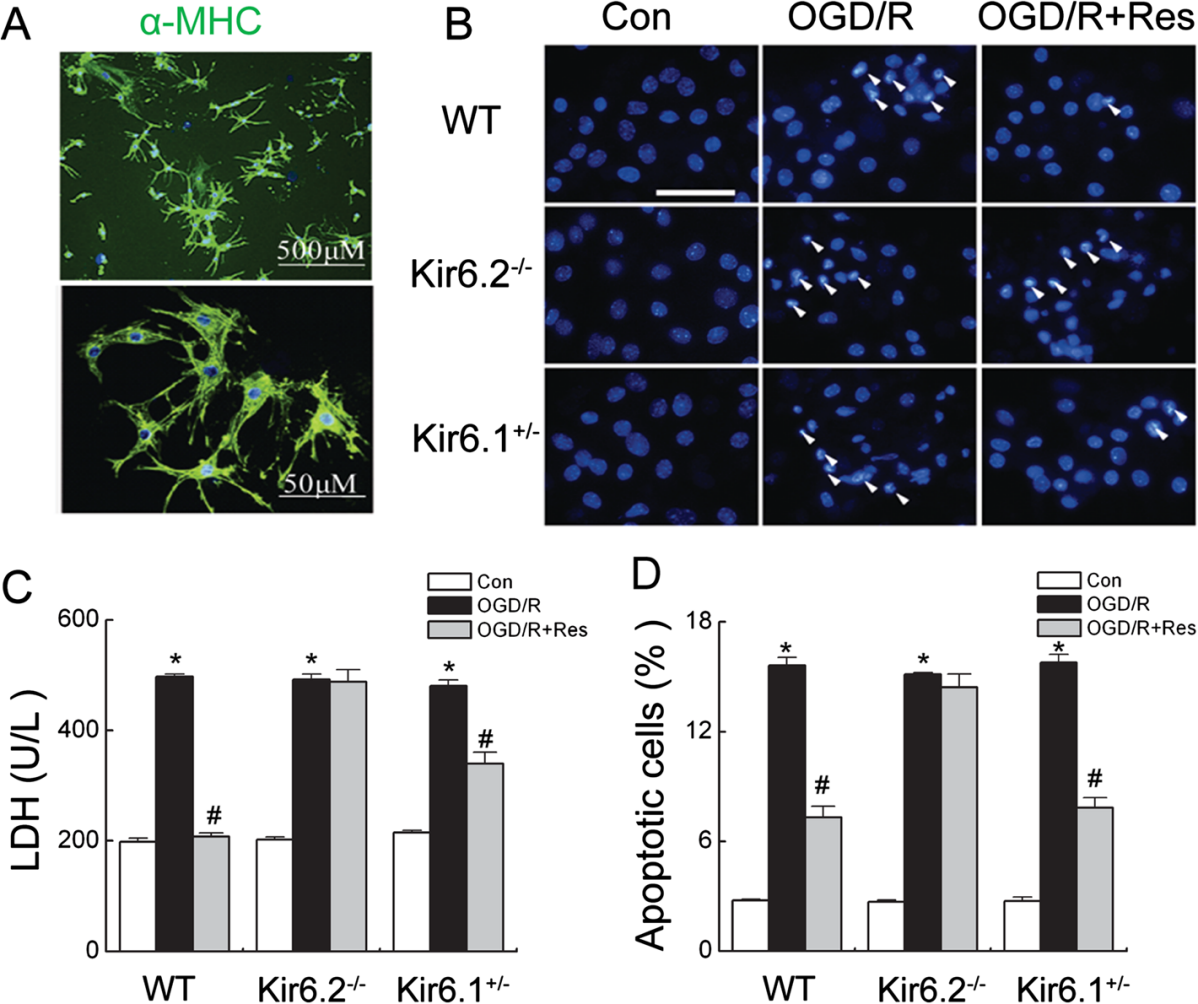
**Kir6.2 knockout abolished the protection of resveratrol against OGD/R injury in cardiomyocytes. (A)** Representative photomicrographs of cardiomyocytes stained with anti-α-myosin heavyweight chain monoclonal antibody. **(B-D)** The cardiomyocytes were pretreatment with resveratrol for 30 min following subjected to 30 min OGD plus 24 hours of reoxygenation. OGD/R induced cell injuries were assessed by staining with Hoechst 33342 **(B, D)** and measuring LDH release **(C)** (n = 6). Data are represented as means ± SEM from six independent experiments. ^*^P < 0.01 *vs*. corresponding control group; ^#^*P <* 0.01 *vs*. corresponding OGD/R group. Arrows point to apoptotic nuclei, Scale bar: 100 μm.

Cardiomyocytes identified by α-myosin heavyweight chain (α-MHC) monoclonal antibody (Figure 3A*)* were cultured to analyze the protective effects of resveratrol *in vitro*. Cardiomyocytes were exposed to 3 h of OGD plus 24 h of reoxygenation in the presence or absence of resveratrol, and cell apoptosis was assessed with Hoechst33342 and cell injury was reflected by LDH release. OGD/R remarkably increased the number of apoptotic cells and LDH leakage in each genotype. However, no significant difference was observed in the number of apoptotic cells among WT, Kir6.2^-/-^ and Kir6.1^+/-^ groups. Resveratrol (10 μM) markedly inhibited OGD/R-induced cell apoptosis and LDH leakage in WT and Kir6.1^+/-^ groups (Figure 3B-D). But, resveratrol failed to reduce the number of apoptotic cells and LDH release in Kir6.2^-/-^ group (Figure 3B-D). These results demonstrate that Kir6.2/K-ATP channels are required for the cardioprotection of resveratrol in cardiomyocytes.

### AMPK - Kir6.2/K-ATP channel signal pathway mediates cardioprotection of resveratrol

**Figure 4:**
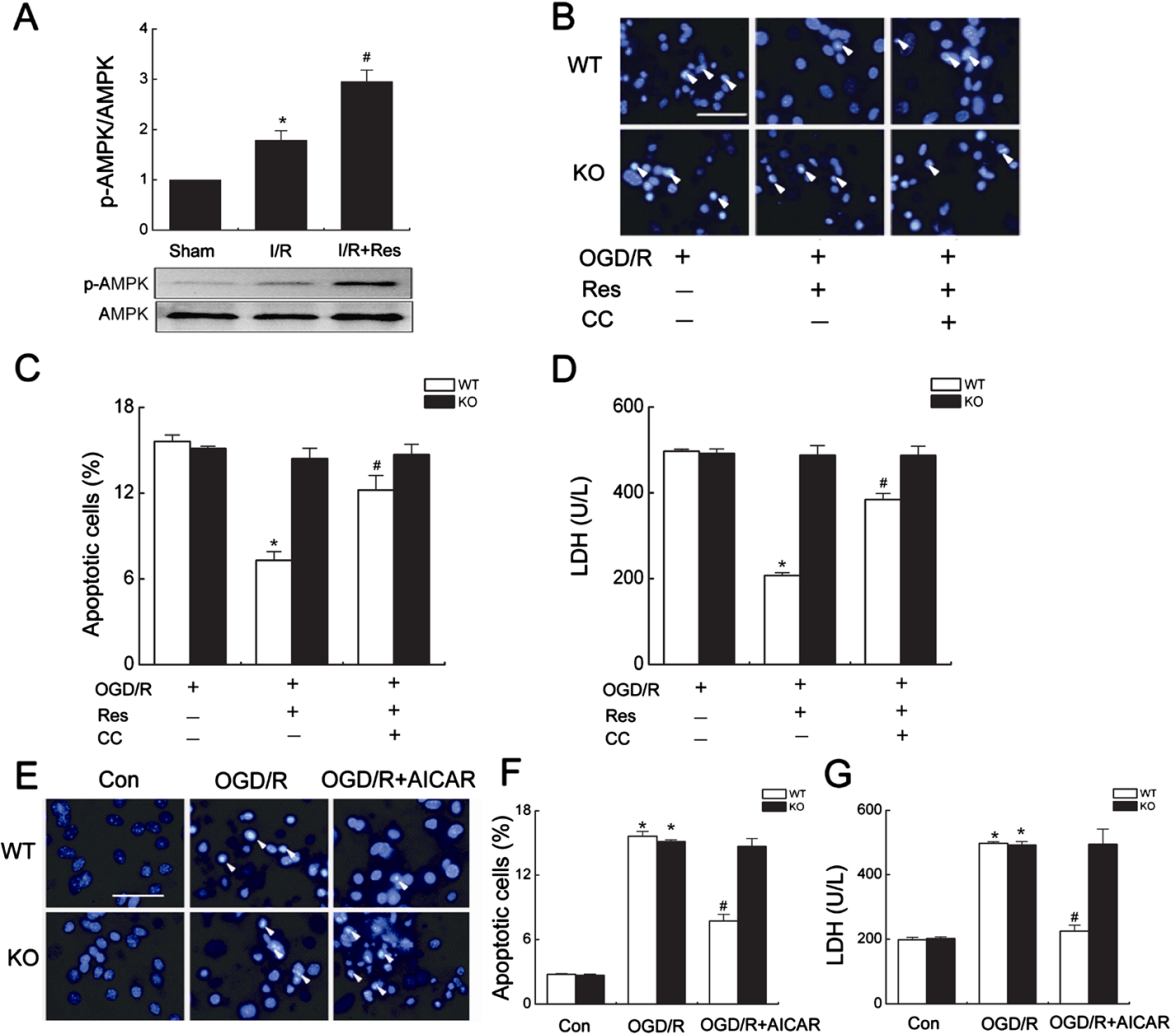
**AMPK-Kir6.2/K-ATP channel mediated cardioprotection of resveratrol. (A)** Representative immunoblots and quantification of myocardial protein extracts for analysis of total AMPK and p-AMPK obtained from wild type (WT) hearts (n = 6/group). ^*^P < 0.05 *vs*. WT sham group, ^#^*P <* 0.05 *vs*. WT I/R group. **(B-D)** After preincubation with Compound **C** (CC, 10 μM) for 30 min followed by resveratrol (Res, 10 μM) 30 min, cardiomyocytes were subjected to OGD device. Cell injuries were assessed by staining with Hoechst 33342 **(B-C)** and measuring LDH release **(D)**. Scale bar: 100 μm. Arrows point to apoptotic nuclei. Data are represented as means ± SEM from six independent experiments. ^*^P < 0.05 *vs.* corresponding OGD/R group; ^#^*P <* 0.05 *vs.* corresponding OGD/R + Res group. **(E-G)** WT and Kir6.2^-/-^ (KO) myocytes were treatment with AICAR (50 μM) following subjected to OGD/R device. Cell injuries were assessed by staining with Hoechst 33342 (E-F) and measuring LDH release **(G)**. Data are represented as means ± SEM from six independent experiments ^*^P < 0.01 *vs*. corresponding control group; ^#^*P <* 0.01 *vs*. corresponding OGD/R group.

Activation of AMPK in the heart protects cardiac ischemic injury [17]. Resveratrol treatment significantly enhanced AMPK phosphorylation in the ischemic heart following reperfusion (Figure 4A), suggesting that AMPK may be involved in cardioprotective effects of resveratrol. To further identify the roles of AMPK in cardioprotection of resveratrol, cardiomyocytes were treated with AMPK inhibitor compound C and AMPK activator AICAR. Suppression of AMPK markedly attenuated the protective effects of resveratrol against OGD/R injury in WT cardiomyocytes as determined by cell apoptosis and LDH leakage (Figure 4B-D). In contrast, activation of AMPK mimicked the cardioprotective effects of resveratrol as confirmed by decreased OGD/R-induced cell apoptosis and LDH leakage in WT cardiomyocytes (Figure 4E-G). Most notably, Kir6.2 knockout reversed cardioprotective effects of AMPK activation (Figure 4E-G), suggesting AMPK might be an upstream of Kir6.2/K-ATP channels. Together, these results indicate that resveratrol promotes the survival of cardiacmyocytes via activation of AMPK - Kir6.2/K-ATP channel signal pathway.

### Resveratrol enhances the association of AMPK with Kir6.2 in cardiomyocytes

**Figure 5:**
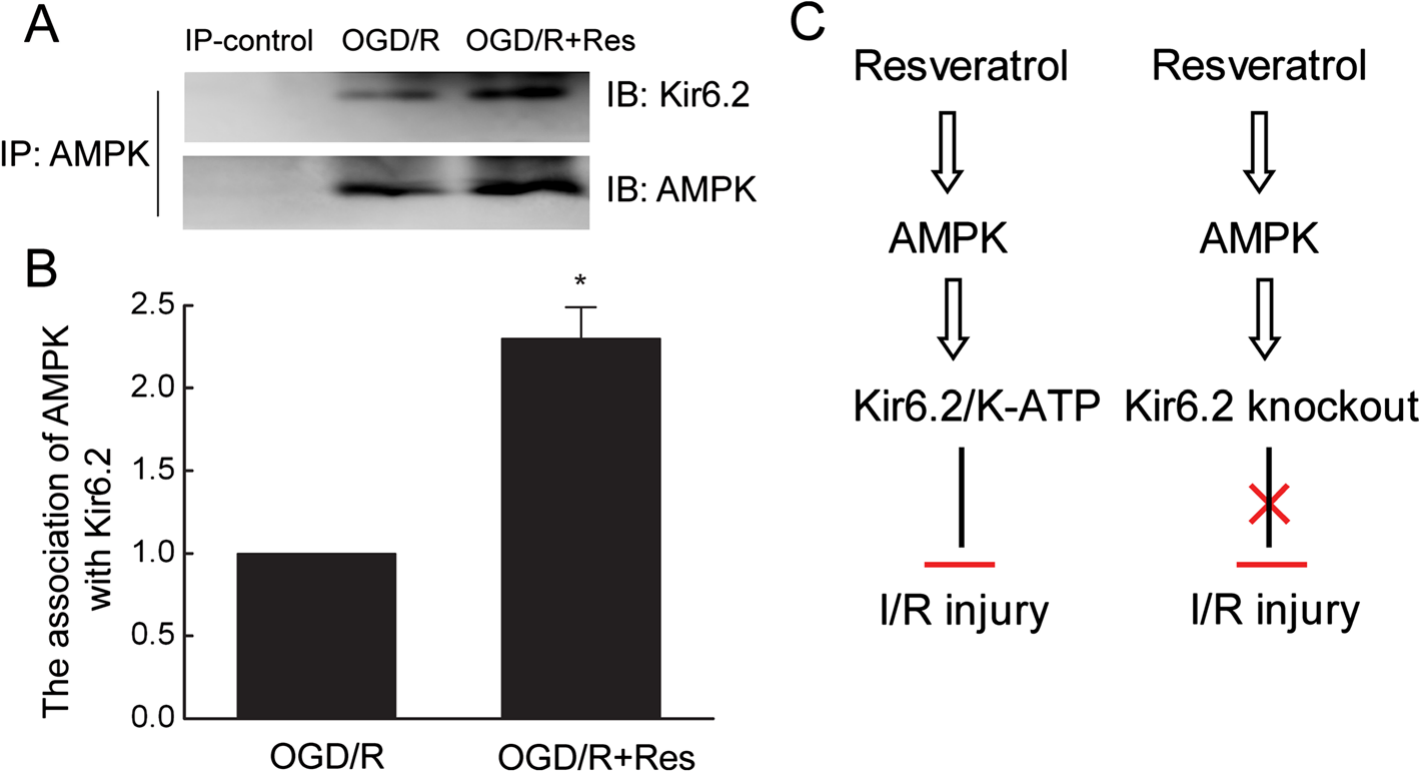
**Resveratrol enhanced the association of AMPK with Kir6.2 in cardiomyocytes. A-B**, The interaction of Kir6.2 and AMPK was analyzed by immunoprecipitation and western blot analysis in cardiomyocytes in the presence of resveratrol. Data are represented as means ± SEM from four independent experiments ^*^P < 0.05. **C**, Schematic layouts of showing Kir6.2/K-ATP channels are required for the protection of resveratrol against ischemia/reperfusion injury.

It has been reported that AMPK mediates preconditioning in cardiomyocytes by regulating the activity and recruitment of sarcolemmal K-ATP channels [18]. To investigate the possible mechanism of AMPK regulating Kir6.2/K-ATP channel in resveratrol – mediated the cardioprotection, the co-immunoprecipitation experiment was performed using an anti-AMPK antibody in cardiomyocytes. As shown in Figure 5 A-B, AMPK was found to interact with Kir6.2, and resveratrol treatment led to a significant increase in Kir6.2-AMPK association. These results suggest that resveratrol protects against cardiacmyocyte apoptosis by enhancing the association of Kir6.2 with AMPK.

## Discussion

In the present study we demonstrated that Kir6.2 knockout abolished the protection of resveratrol against myocardial ischemia/reperfusion injury *in vivo and in vitro*. Resveratrol enhanced the activity of AMPK and promoted the association of AMPK with Kir6.2. Suppression of AMPK attenuated the cardioprotection of resveratrol whereas activation of AMPK mimicked protective effects of resveratrol. In addition, Kir6.2 knockout reversed the cardioprotective effects of AMPK activation. Thus, Kir6.2/K-ATP channel is required for resveratrol-mediated protection against myocardial ischemia/reperfusion injury and AMPK-Kir6.2/K-ATP channel signal pathway mediates cardioprotection of resveratrol (Figure 5C).

K-ATP channel is a kind of molecular sensors that plays a critical role in cardiovascular adaptive response under the challenge of stress [19]. The cardiac K-ATP channels are composed of Kir6.2 subunits in combination with SUR2A [20], while Kir6.1 subunit is a major molecular component of vascular smooth muscle K-ATP channels [21]. Opening K-ATP channels blocks cardiomyocyte apoptosis in ischemia-reperfusion [22]. However, the specific roles of Kir6.1 and Kir6.2 in myocardial protection remain unclear. As Kir6.1 knockout mice could not endure I/R injury (the mortality rate within 24 hours after I/R injury was 100%), Kir6.1^+/-^ mice and Kir6.2 knockout mice were used to subject to I/R injury in the present study. We found that the infarct size was much larger in Kir6.1^+/-^ mice compared to WT mice following myocardial I/R injury. But, no significant difference was detected in isolated cardiomyocyte apoptosis after OGD/R treatment between WT and Kir6.1^+/-^ mice. Kir6.1/K-ATP channel has been demonstrated to be critical in the regulation of vascular tone, especially in the coronary arteries [23] . As a result, more severe damage in Kir6.1^+/-^ mice induced by I/R injury might be due to vasodilatation disorder, instead of cardiomyocyte dysfunction. Furthermore, there were no significant differences in infarct size between the WT and Kir6.2^-/-^ mice after I/R injury, which was consistent with the report of Masashi Suzuki [24].

Recent studies suggest that resveratrol enhances activity of the K-ATP channels in rat hearts [25] and activation of the K-ATP channel is necessary for resveratrol action [26]. As opposed, the experiments on the mouse beta cell line revealed that resveratrol blocked K-ATP channels [27] by directly binding to sulfonylurea receptor 1 (SUR1) of K-ATP channel [28]. This discrepancy in the results obtained on the various experimental models strongly suggests tissue and species specificity of resveratrol. In addition, pancreatic β-cell K-ATP channels are thought to be composed of Kir6.2 and SUR1 subunits [29], and it is plausible that SUR1 may not be primarily expressed in ventricular myocytes [30]. Resveratrol is specific to SUR1 subunits, and thus it causes opening of Kir6.2 SUR2A K-ATP channel in cardiac tissues but blocks Kir6.2 SUR1 K-ATP channel in pancreatic tissues. It is not immediately clear how to reconcile these different outcomes, and further work may be needed to determine whether there is a differential mechanism involved.

Emerging evidence indicates that resveratrol protects cardiomyocytes from I/R injury via a combination of suppression of superoxide levels and activation of potassium channels [7]. However, the relation of K-ATP channel and resveratrol in I/R injury remains unknown. Thus, the aim of this study was designed to assess the roles of Kir6.2 or Kir6.1–containing K-ATP channel in cardioprotection of resveratrol under I/R injury. Our work found that resveratrol reduced the infarct size and decreased the activity of serum LDH and CK following I/R injury, and Kir6.2 knockout abolished the cardioprotective effects of resveratrol *in vivo*. In addition, Kir6.2 knockout also reversed the suppressive effects of resveratrol on cardiomyocyte apoptosis after OGD/R treatment *in vitro*. But, Kir6.1 knockdown failed to abolish the cardioprotection of resveratrol. These findings indicate that Kir6.2/K-ATP channel is required for resveratrol-mediated cardioprotection.

It is well known that the most prominent role of K-ATP channels in cardiovascular system is that opening of this channel can protect cardiac myocytes against ischemic injuries. K-ATP channels are present in multiple tissues and cell types within the cardiovascular system and these channels differ from each other in terms of their biophysical and pharmacological properties. The properties of K-ATP channels are different in various tissues due to the combinations of the subunits forming the channel. The “cardiac” K-ATP channel has conventionally been thought to consist of Kir6.2/SUR2A subunits. Although SUR1 subunits are not essential subunits of ventricular sarcolemmal K-ATP channels, the evidence is strong that SUR1 subunits are also expressed in the heart (particularly in atria) and that they may have a functional role. In contrast to Kir6.2-null and SUR2-null mice, SUR1-null mice are protected from ischemia/reperfusion [31]. One interpretation is that SUR1 subunits might actually contribute to ischemic or post-ischemic damage. SUR1 levels are up-regulated after ischemic events; even in tissues where it is not normally expressed, such as the endothelium [32]. Recent data suggest that SUR1 subunits associate with TRPM4 subunits [33], which may modulate the membrane potential and ionic gradients, thus contributing to post-ischemic injury. It was recently demonstrated that resveratrol is a natural SUR1 ligand that can induce apoptosis in a SUR1 isoform-specific manner [28]. One might predict that resveratrol reduces infarct size after ischemia/reperfusion by blocking the cardiovascular SUR1-containing channels.

Resveratrol provides cardioprotection by triggering different endogenous signaling pathways including oxidative stress/antioxidant defense system, glucose/insulin metabolism, iNOS/nitrotyrosine, and preconditioning, which are associated with energy metabolism [34-36]. AMPK is an energy sensor protein that is activated in response to ATP depletion [37]. AMPK activation plays a critical roles in ischemia/reperfusion injury in the heart [38,39]. A recent report demonstrated that resveratrol protected ROS-induced cell death by activating AMPK in the H9c2 cardiac muscle cell line [40]. In the present study, resveratrol enhanced AMPK activation in ischemic heart. Blockade of AMPK activity abolished the suppressive effects of resveratrol on cardiomyocyte apoptosis. Moreover, activation of AMPK mimicked protective effect of resveratrol on survival of cardiomyocytes. Taken together, these data indicate that resveratrol can exert beneficial actions on the cardiovascular systems partly through the AMPK-dependent mechanism. Furthermore, recent study has showed that AMPK mediates preconditioning in cardiomyocytes by regulating the activity and recruitment of sarcolemmal K-ATP channels, but the exact mechanism is still unclear [18]. In our study, we found that AMPK interacted with Kir6.2 and resveratrol increased the association of Kir6.2 with AMPK. This interaction may trigger and promote Kir6.2/K-ATP channel opening, which mediates cardioprotection of resveratrol. Notably, Kir6.2 knockout abolished the cardioprotection of AMPK activation, indicating that Kir6.2/K-ATP channel might be downstream of AMPK. Thus, resveratrol enhances the AMPK phosphorylation, which in turn increases the association of AMPK with Kir6.2, and then promotes Kir6.2/K-ATP channel opening, subsequently protects against I/R injury.

## Conclusions

Resveratrol exerts cardioprotective effects through AMPK -Kir6.2/K-ATP signal pathway and Kir6.2-containing K-ATP channel is required for cardioprotection of resveratrol. Modulation of the energy metabolic pathway via AMPK or Kir6.2/K-ATP could be a novel cardioprotective strategy for I/R injury.

## Competing interests

The authors declare that they have no competing interests.

## Authors’ contributions

R-H D and TD designed and executed the experiments, interpreted data, performed the statistical analysis and wrote the manuscript. W-J C, ML and J-h D performed animal experiment. GH conceived the study, and participated in its design and helped to draft the manuscript. All authors read and approved the final manuscript.
